# A new chapter for FEBS Open Bio

**DOI:** 10.1002/2211-5463.12781

**Published:** 2020-01-05

**Authors:** Miguel A. De la Rosa

## Abstract

In this Editorial, the new Editor‐in‐Chief Professor Miguel A. De la Rosa introduces his plans for the journal.

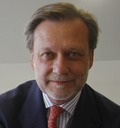

This issue marks a new chapter in the 8‐year history of *FEBS Open Bio*, as it is with enormous pride that I announce the beginning of my tenure as Editor‐in‐Chief. Since its inception in November 2011, *FEBS Open Bio* has been under the helm of Mary Purton as Executive Editor, who has guided the journal to maturity and great success. *FEBS Open Bio* has recently published its 1000^th^ article 1, and submissions to the journal continue to grow at a tremendous pace. To meet the ever‐increasing demands of operating the journal, FEBS has decided to divide the Executive Editor position into two separate roles, appointing me as Editor‐in‐Chief and hiring a full‐time Editorial Manager. Mary Purton has been appointed as FEBS Press Publisher and will coordinate editorial activities across *FEBS Open Bio*, *The FEBS Journal*, *FEBS Letters* and *Molecular Oncology*. I wish her great success in this important role, and I look forward to working with her to ensure the journal retains its high standards.

As Editor‐in‐Chief, I will be working closely with the journal’s prestigious editorial board to ensure that we continue to meet the journal’s mission of publishing technically sound manuscripts. I also intend to appoint new editors to ensure that the journal is well placed to expertly review manuscripts on research areas across the life sciences, as befits a multidisciplinary mega journal. In particular, I hope to increase editorial representation from Latin America, Asia and Eastern Europe, to ensure that the board continues to represent the scientific community. Our editorial board will be supported by staff in our editorial office in Cambridge: I would like to welcome Duncan Wright as our new Editorial Manager, who will be responsible for managing the day‐to‐day operation of the journal. Duncan and I will continue to be ably supported by Jacob Weller in his role as Editorial Assistant. I would like to extend my thanks to all of our editors and the editorial office staff for their hard work on the journal, which is instrumental to the journal’s success.

Downloads for the journal have increased dramatically over the last year, with downloads in October 2019 80% greater than the same month in 2018. Our most downloaded article ever is a Method paper published last year, which describes a protocol for rapid bacterial identification based on sequencing of 16S rRNA genes 2. Our other most highly downloaded articles cover a breadth of research fields, including optimization of fluorescent protein expression for fluorescence microscopy 3, a report that colonization by *Bifidobacterium infantis* may help maintain barrier function in the gastrointestinal tract of infants 4, and a report that metformin inhibits neuroinflammation in mice with diet‐induced obesity 5. This trend towards increased downloads emphasizes that the journal’s publications are of interest and use to the scientific community. In addition, many of the articles we publish are highly cited; our most highly cited paper was published in 2014 and has received 189 citations at the time of writing 6.

In 2017, FEBS Open Bio started an Education section, publishing original research articles on pedagogical approaches in biochemistry and molecular biology. To date, we have published six Education articles covering a range of topics, including monitoring the effect of changing teaching practices on student performance in an introductory biochemistry course 7, the effect of the use of clickers in lectures 8 and a comparison of selected doctoral training programmes in the UK and Scandinavia 9. One of the journal’s most downloaded papers is a study of the relationship between student seat preference in the lecture hall and educational attainment 10; this paper is also in the top 5% of all research outputs scored by Almetric, indicating that this paper garnered a high level of attention online. The Education section is edited by Angel Herráez and Luciane Mello, and I am grateful for their continued hard work and dedication to this important component of the *FEBS Open Bio* mission.

Finally, as part of my long‐term vision for the journal, I intend to increase ties between *FEBS Open Bio* and the annual FEBS Congress (to be held this year in Ljubljana, Slovenia) through various new initiatives. It is my hope that *FEBS Open Bio* will serve as the official journal of the Congress and that this will facilitate mutual promotion between the various activities of FEBS. I look forward to introducing this and other new developments over the upcoming year. On the behalf of the *FEBS Open Bio* editorial board and office staff, I wish all of our readers, authors and reviewers a happy and productive new year, and I hope you will continue to consider us when publishing your work.
